# Healthcare Use during the Last Six Months of Life in Patients with Advanced Breast Cancer

**DOI:** 10.3390/cancers13215271

**Published:** 2021-10-20

**Authors:** Renée. S. J. M. Schmitz, Sandra. M. E. Geurts, Khava. I. E. Ibragimova, Dominique. J. P. Tilli, Vivianne. C. G. Tjan-Heijnen, Maaike de Boer

**Affiliations:** Department of Internal Medicine, Division of Medical Oncology, GROW-School for Oncology and Developmental Biology, Maastricht University Medical Center+, 6229 HX Maastricht, The Netherlands; r.schmitz@nki.nl (R.S.J.M.S.); sandra.geurts@mumc.nl (S.M.E.G.); khava.ibragimova@mumc.nl (K.I.E.I.); dominique.tilli@mumc.nl (D.J.P.T.); vcg.tjan.heijnen@mumc.nl (V.C.G.T.-H.)

**Keywords:** breast neoplasms, neoplasm metastasis, end of life, delivery of health care, chemotherapy, SONABRE Registry

## Abstract

**Simple Summary:**

In the last decades, new treatment options for advanced (breast) cancer have resulted in increased use of health care resources near the end of life. We assessed health care use near the end of life of patients with advanced breast cancer (ABC). In this study, we have shown that ICU admission, and CPR occurred rarely during the last six months of life of ABC patients. However, hospital admissions occurred often, especially in patients who received new chemotherapy within 30 days of end of life. Those patients were also more likely to die in the hospital. However, death was most often due to disease progression. To improve quality of life near the end of life of advanced breast cancer patients, it is vital to develop tools to help clinicians identify those patients who will benefit from chemotherapy at the end of life.

**Abstract:**

New treatment options in cancer have resulted in increased use of health care resources near the end of life. We assessed health care use near the end of life of patients with advanced breast cancer (ABC). From the Southeast Netherlands Breast cancer (SONABRE) registry, we selected all deceased patients diagnosed with ABC in Maastricht University Medical Center between January 2007 and October 2017. Frequency of health care use in the last six months of life was described and predictors for health care use were assessed. Of 203 patients, 76% were admitted during the last six months, 6% to the intensive care unit (ICU) and 2% underwent cardiopulmonary resuscitation (CPR). Death in hospital occurred in 25%. Nine percent of patients received a new line of chemotherapy ≤30 days before death, which was associated with age <65 years and <1 year survival since diagnosis of metastases. In these patients, the hospital admission rate was 95%, of which 79% died in the hospital, mostly due to progressive disease (80%). In conclusion, the frequency of ICU-admission, CPR or a new line of chemotherapy ≤30 days before death was low. Most patients receiving a new line of chemotherapy ≤30 days before death, died in the hospital.

## 1. Introduction

Breast cancer is the most common type of cancer in the western world and the number one cancer-related cause of death among women worldwide [1,2,3]. During the last 25 years, much progress has been made in the treatment of advanced (i.e., metastatic) breast cancer (ABC). Median overall survival rates differ per tumor subtype [4], and in general, increased from 13 months 1985–1990 to 33 months in 2010–2014 [5]. The introduction of new targeted therapies, including CDK4/6 inhibitors for HR+/HER2− disease [6,7] and trastuzumab, pertuzumab [8] for HER2+ disease have led to an increase in overall survival in ABC. 

Although at first palliative treatments may be effective, advanced (breast) cancer will eventually become therapy resistant and will result in death. In studies in patients with advanced cancer in general, novel treatment options have resulted in increased use of healthcare resources near the end of life [9], such as admission to the hospital [10,11], intensive care unit (ICU) admission [10,11,12], resuscitation (CPR), mechanical ventilation, radiotherapy, surgery, chemotherapy [6,7,8,10,12,13] and death in the hospital [10,12,13]. 

The challenge is to balance between using available health care resources and choosing what is suitable in the situation facing the end of life while aiming for optimal quality of life (QoL). Prigerson et al. observed that chemotherapy use near the end of life did not improve QoL in patients with moderate or poor performance status, and actually worsened QoL in patients with a good performance status [14]. However, in daily practice, predicting the patient’s end of life and identifying whether a patient with ABC will benefit from another line of chemotherapy is difficult [15]. The identification of factors related to chemotherapy use and other health resources near the end of life may improve shared decision-making on whether or not to start or continue antitumor treatment. Thus, it may improve end of life care and QoL in patients with ABC. However, there is little information on the specific use of healthcare resources for patients with specific cancer types, and breast cancer in particular, during the end of life. In the current study, we assessed the use of health care resources near the end of life in ABC patients in our university hospital and identified factors related to the use of these resources.

## 2. Materials and Methods

### 2.1. Patients

From the SOutheast Netherlands Advanced BReast Cancer (SONABRE) Registry (NCT-03577197), we selected all deceased patients from Maastricht University Medical Center (MUMC+) who were aged 18 years or older and who were diagnosed with ABC between 1 January 2007 and 1 October 2017. The data lock was on 23 October 2017. The Medical Research Ethics Committee of Maastricht University Medical Centre approved the registry (15-4-239).

### 2.2. Data Collection

Patient, disease, and treatment characteristics from the diagnosis of breast cancer until death were collected from the medical files by specially trained registration clerks. In addition, type and reason of (ICU) admissions, frequency of CPR, and mechanical ventilation were obtained for the final six months of life, as were the date, cause, and place of death. 

### 2.3. Definitions

Estrogen (ER) and progesterone receptor (PR) positivity was defined as positive nuclear staining of ≥10% and hormone receptor (HR) positivity was defined as ER and/or PR positive breast cancer. HER2 positivity was defined as an immunohistochemistry score of 3+, or 2+ with a positive fluorescence in situ hybridization (FISH) result. Tumors were subdivided in HR+/HER2− (including HR+/HER2 unknown), HR+/HER2+, HR−/HER2+, and HR−/HER2− disease. If available, biopsy information of the initial metastatic lesion was used, alternatively, biopsy information of a recurrence or the primary tumor were used. *De novo* ABC was defined as diagnosis of metastasis <3 months after diagnosis of primary disease [16]. The definition of chemotherapy was chemotherapy, or chemo-targeted therapy, such as but not limited to paclitaxel-bevacizumab or docetaxel-trastuzumab. Endocrine-targeted therapy was defines as endocrine therapy (tamoxifen, aromatase-inhibitor or fulvestrant) combined with targeted therapy such as everolimus, trastuzumab, or bevacizumab. Endocrine therapy consisted of tamoxifen, aromatase-inhibitor, fulvestrant or megestrol. Targeted therapy consisted of trastuzumab, palbociclib or brivanib (in a clinical trial).

### 2.4. Outcomes

End of life was defined as the period of the last six months of life [17]. The following health care procedures were described: ongoing chemotherapy ≤14 days before death, start of a new line of chemotherapy ≤30 days before death, emergency room (ER) visit, admission to the hospital, intensive care unit (ICU) admission, cardiopulmonary resuscitation (CPR), mechanical ventilation, surgery, radiotherapy, and place of death [9,18]. For patients who died in the hospital, the hospital ward of death was described. Furthermore, the association of these health care resources and death in the hospital between incidence year (2007–2009, 2010–2013, 2014–2017), age at diagnosis (<65 years vs. 65+ years), subtype (HR+/HER2−, HR+/HER2+, HR−/HER2+, HR−HER2−), metastatic-free interval (MFI) (*de novo*, 3–24 months, >24 months), WHO performance status at start last treatment line (0–1, 2–4), and survival time (i.e., time between diagnosis of ABC and death; <1, ≥1 year) was assessed. 

### 2.5. Statistics

Descriptive statistics were used to describe the frequency of health care use. Multivariable logistic regression analyses were performed to assess predictors for the use of these health care resources and death in the hospital. For the regression analysis of factors associated with new chemotherapy use ≤30 days before death, or ongoing chemotherapy ≤14 days before death, all patients not having received chemotherapy (never or no new line ≤30 days before death or ongoing chemotherapy ≤14 days before death) were the control group. Patients with unknown subtype (*n* = 3) were excluded from the multivariable analyses, and values of patients with unknown WHO performance status (*n* = 18) were imputed. A *p*-value of ≤0.10 was considered as borderline significant, and ≤0.05 as statistically significant.

## 3. Results 

From 1 January 2007 until 1 May 2017, 327 patients were diagnosed with ABC in MUMC+. Of those, 211 patients had died before the data lock on 23 October 2017. In total, eight patients were excluded because of insufficient data (*n* = 4) or lost to follow-up (*n* = 4). The remaining 203 patients were included in the analyses. 

Table 1 describes the patient, disease, and treatment characteristics of all included patients, and is specified for patients who died in the hospital (*n* = 50). Of the total population, all patients were female, the median age at diagnosis was 63 years, 61% of patients were diagnosed with HR+/HER2−, 14% with HR+/HER2+, 10% with HR−/HER2+, 13% with HR−/HER2− disease, and subtype was unknown for 2%. Patients received a median of 2 treatment lines for ABC, where 10% of the patients did not receive any palliative systemic therapy. Table S1 describes the final systemic therapies which were given before death. Of the patients who died in the hospital, 34% had de novo metastatic disease versus 26% in the total ABC population, 64% received chemo(-targeted) therapy as final systemic therapy versus 54% in the total population, death due to complications of systemic therapy occurred in 16% versus 4% for the total population, and in 2% time from diagnosis of ABC to death was ≥4 years versus 15% for the total population.

Concerning the frequency of health care use, 9% of patients (*n* = 19) received a new line of chemotherapy within the last 30 days of life (Table 2). During the last 14 days prior to death, 21% of patients received an ongoing line of chemotherapy. During the last six months of life, 76% of patients were admitted to the hospital at least once (in 60% of those, due to tumor-related symptoms), 6% were admitted to the ICU (50% because of tumor related symptoms), and 2% underwent CPR. Most patients died at home (53%), followed by 25% who died in the hospital, 14% in a hospice, 3% in a rehabilitation center, and for 6% of patients location of death was unknown after discharge from the hospital. Eighty percent of all patients who died in the hospital died in the general ward, 18% in the intensive or medium care unit, and 2% in the ER. 

In multivariate analyses, HR+/HER2+ disease was related to fewer admissions ≤ six months before death (62% admissions versus 75%, 85% and 90% for HR+/HER2−, HR−/HER2− and HR−/HER2+, respectively, OR = 0.29 (95%CI:0.11–0.81)). Related to more admissions ≤ six months before death were age at diagnosis of ABC <65 years (84% admission versus 67% in patients ≥65 years, OR = 3.12 (95%CI:1.37–7.05)); de novo metastatic ABC (89% admissions versus 75% and 70% for 3–24 months and >24 months metastatic free interval, OR = 4.41 (95%CI:1.55–12.5)); survival time <1 year (81% admissions versus 73% for survival time >1 year, OR = 2.41 (95%CI:0.97–5.99)); and ongoing chemotherapy ≤14 days before death (95% admissions versus 71% for no ongoing chemotherapy, OR = 12.8 (95%CI:1.38–120)) (Table 3). New chemotherapy ≤30 days before death was related to death in the hospital (79% versus 18% for no new chemotherapy, OR = 14.4 (95%CI:2.85–73.0)) (Table 3) in multivariate analyses. Of the 19 patients receiving a new line of chemotherapy ≤30 days before death, 18 (95%) were admitted at least once ≤ six months before death: 4 due to tumor progression, followed by new chemotherapy administration during that admission, and 14 patients were admitted after new chemotherapy administration (≤30 days before death) because of toxicity (*n* = 4) or tumor-related symptoms (*n* = 10). Death was due to toxicity in 16% and tumor progression in 84% of those 19 patients. Of the patients who died in the hospital after a new chemotherapy administration (≤30 days before death), 20% was due to toxicity and 80% due to tumor progression (data not shown). 

The following characteristics were related to start of a new line of chemotherapy ≤30 days before death: lower age (<65 vs. ≥65 years, OR = 11.8 (95%CI:2.46–56.9)); and survival time <1 year (vs. survival time ≥1 year, OR = 4.50 (95%CI:1.40–14.5)) (Table 4). Lower age (<65 vs. ≥65 years, OR = 4.76 (95%CI:2.04–11.1), *p* < 0.01), and HR−/HER2− subtype (vs. HR+/HER2− subtype, OR 3.65 (1.36–9.80) were independently associated with a higher rate of ongoing chemotherapy ≤14 days before death (Table 4).

## 4. Discussion

In this observational study, we assessed health care use during the last six months of life in patients with ABC who were diagnosed and had died within 10 years. We observed that 76% of the patients were hospitalized within the last six months before death, and 25% of patients died in the hospital. Admission to ICU (6%), mechanical ventilation (5%), and CPR (2%) occurred infrequently. Nine percent of patients started a new line of chemotherapy ≤30 days before death, and 21% of patients continued chemotherapy within 14 days before death. Overall, 79% of patients receiving a new line of chemotherapy within 30 days before death, died in the hospital. Likewise, half of the patients receiving ongoing chemotherapy within 14 days before death, died during hospital admission. 

Definitions of intensive use of health care resources at the end of life vary widely, although the most commonly reported measurable outcomes across studies were admissions to ICU; administration of CPR; and initiation or continuation of chemotherapy in the last 14 to 30 days of life [9]. In a (non-breast) metastatic cancer study analyzing the association between high-risk clinical events and survival, unplanned hospitalization was associated with higher mortality [19]. Ferrario et al. reported an increase in ICU admissions within 30 days from death from 14% in 2000–2003 and 23% in 2010–2014, and an increase in >1 admissions within 30 days from death from 11 to 14% in commercially insured metastatic breast cancer patients aged <65 years in the USA [20]. We found a high admission rate of 76% within six months before death. In a longer time frame of 12 months in an ABC population, a 90% admission rate was reported by Tanguy et al. [21]. We observed that age <65 years, de novo metastatic breast cancer, and a survival time <1 year were associated with hospital admission(s) within six months before death. Conversely, HR+/HER2+ disease was related to less frequent admissions. As symptom relief was the most frequent reason for admission, we can hypothesize that patients of lower age, patients with de novo metastatic disease, or patients with shorter survival time more often have symptomatic disease for which in-hospital symptom relief is necessary. We cannot explain the reason for less frequent admissions in the HR+/HER2+ subgroup. Although the overall ICU admission rate was low (6%) in our study, we observed an ICU admission rate of 26% in patients after receiving chemotherapy ≤30 days before death. This association was also seen in another general cancer study [22]: 8% ICU admissions within 1 week before death in patients not receiving chemotherapy versus 14% in patients who received chemotherapy in the last six months of life. Severe toxicity of chemotherapy can explain part of the ICU admissions, although half of the patients admitted to ICU were admitted because of symptomatic disease. 

At least two-thirds of people prefer a home death in all but one of seven European countries studied by Gomes et al. [23]. This strong association with personal values suggests keeping home care at the heart of cancer end of life care [23]. In a cross-national European end of life study in cancer and non-cancer patients from general practitioners, hospital death rates were lowest for Dutch patients (28%), and highest for Italian patients (39%) [24]. We reported a hospital death rate of 25% (4% in the ICU), comparable to the rate in a recent Dutch insurance data study in upper gastro-intestinal (GI) cancer patients of whom 23% died in the hospital [25]. In metastatic breast cancer, one French and one US study reported in-hospital death rates of 70% [21] and 19% [26], respectively. The hospital death rate can be influenced by the availability of home care and hospice services, which differ between countries [27,28].In our study, we confirmed the association as reported by several others [9,18,21] between the initiation of a new line of chemotherapy within 30 days before death and death in the hospital, with a hospital death rate of 79% for patients receiving chemotherapy versus 18% for those who did not. In these patients, cause of death was more often due to toxicity (16%) as compared to the total population (4%), although most patients died of progressive disease. 

In our study, 9% of ABC patients received a new line of chemotherapy in the final 30 days of life and 21% of the patients received ongoing chemotherapy in the final 14 days of life. Several other breast cancer studies have reported the rate of chemotherapy administration within 14 days before death, ranging from 10% to 24% in US, French and Swedish populations [20,21,29,30] and 49% in a Greek ABC population [29]. Our rate of new chemotherapy within 30 days before death was comparable to findings in a Swedish population (8%), although in a Greek population, 20% of patients received a new line of chemotherapy [29]. This observation fits in with two studies describing fewer end of life treatment discussions and more use of health care resources including chemotherapy in southern European countries (Italy and Spain) compared to northern European countries (Belgium and The Netherlands) [24,31]. Other factors related to more chemotherapy prescriptions shortly before death in ABC were younger age [29,30], higher albumin levels [29], greater disease burden [30], more prior lines of chemotherapy [30], and in this study we found HR−/HER2− breast cancer, and survival time <1 year as related factors. Larger hospital size was associated with fewer chemotherapy prescription shortly before death in the Dutch GI cancer study [25]. Chemotherapy prescription shortly before death is associated with less palliative care, and more death in the hospital [21,32], the latter also being observed in the current study. Timely addressing the preferences for end of life care in patients with ABC may decrease chemotherapy prescription near the end of life [33]. However, it is challenging to estimate the prognosis of a patient, and select those patients who will not benefit from chemotherapy. 

The goals of treatment of ABC are to prolong survival and improve QoL by reducing cancer-related symptoms. Response rates to palliative chemotherapy are considerable, ranging from 35 to 60% for first-line chemotherapy, and 11 to 35% for third-line chemotherapy [34], which might reflect accurate patient selection. Performance status and benefit from a prior chemotherapy line are the most important predictors of benefit from a new line of chemotherapy [2,35], and jaundice is negatively correlated with benefit from chemotherapy in ABC [2]. The ASCO Choosing Wisely initiative advised not to use cancer-directed therapy for solid tumor patients with the following characteristics: low WHO performance status (3 or 4), no benefit from prior evidence-based interventions, not eligible for a clinical trial, and no strong evidence supporting the clinical value of further anti-cancer treatment [36]. Milton et al. developed a prediction tool to better predict the survival of cancer patients who did not longer receive antineoplastic treatment [37], but these tools do not exist for patients receiving antineoplastic treatment. We did not observe an association between performance status and the use of chemotherapy ≤30 days before death. However, although not statistically significant, we observed that a new line of chemotherapy ≤30 days before death was more frequently started in patients diagnosed with advanced breast cancer in the period 2007–2009 (14%) as compared with 8% in the period 2010–2013, and 3% in the period 2014–2017 (Table 4). This observation corresponds with the increasing awareness regarding predictors for chemotherapy benefit and the importance of end of life care since the last decade, and stresses the importance of continuing education on this topic. Prospective studies in ABC should focus on identifying predictors for benefit of chemotherapy and tools for assessing survival. Then, patients suitable for and willing to receive another line of chemotherapy can be better selected, resulting in less intensive end of life care and better quality of their remaining life for those patients who will not benefit from chemotherapy. In addition to improved patient selection, early access to palliative care for advanced care planning in the phase where a patient with ABC is still on active antineoplastic therapy will improve QoL [38,39].

This is the first study to assess the use of health care resources during end of life in ABC patients in The Netherlands. Compared to other studies in ABC on the use of health care resources, this study is unique as we analyzed not only chemotherapy use or death in the hospital, but also (ICU) admissions and the reason for admission and cause of death. We focused on the use of health care resources in the last six months of life, and chemotherapy use in the last 30 days of life, whereas most other ABC studies focused on health care resource use in the last 30 days of life. We selected patients who died after being diagnosed with ABC in the period 2007–2017. This will have resulted in a bias, as patients with a recent diagnosis were only included in the study if they had died, resulting in a shorter overall survival for this study population as compared to a general ABC population [4]. As the endpoint of the study was not to assess survival, but to determine use of health care resources and factors related to this, we do not think this is a major limitation. Besides the selection criterion of death (from any cause), we did not select patients based on certain characteristics. The real world perspective of this study is reflected by the fact that 10% of the patients did not receive any systemic therapy for advanced breast cancer. This study was conducted in a university hospital, which also serves as a tertiary center for oncology, serving more young patients motivated for trial participation. Therefore, future study in non-academic centers and other countries is required to confirm these results. Due to the retrospective design of the study, performance status was missing for a large proportion of patients. If possible, we estimated the performance status based on the description of the condition by the physician from the file, although this will not have been as accurate as assessment of the WHO performance status by the physician. The identification of predictors and the development of models to help identify those patients who will benefit from another line of chemotherapy is very important although beyond the scope of this study, as we could not assess the causality of relations in this retrospective study. 

## 5. Conclusions

In this study, we have shown that admission to ICU, mechanical ventilation, and CPR rarely occurred in the last six months of life of ABC patients. Conversely, hospital admission occurred often, and a quarter of patients died in the hospital. However, death in the hospital occurred in most patients who received a new line of chemotherapy within 30 days of death, mostly due to disease progression. To improve QoL, more research is warranted to identify those patients who will benefit from chemotherapy at the end of life.

## Figures and Tables

**Table 1:** Patient, disease and treatment characteristics of all patients who died after being diagnosed with advanced breast cancer (left column) and of the 50 patients who died in the hospital (right column).

Variation	All Patients		Patients who Died in Hospital	
	*n* = 203	%	*n* = 50	%
Gender				
Female	203	100%	50	100%
Male	0	0%	0	0%
Incidence year ABC				
2007–2009	81	40%	20	40%
2010–2013	93	46%	24	48%
2014–2017	29	14%	6	12%
Age at diagnosis ABC (years)				
Median (range)	63	(28–90)	63	(28–87)
<65 years	105	52%	28	56%
65+ years	98	48%	22	44%
Subtype				
HR+/HER2−	124	61%	31	62%
HR+/HER2+	29	14%	6	12%
HR−/HER2+	20	10%	5	10%
HR−/HER2−	27	13%	6	12%
Unknown	3	2%	2	4%
Metastatic-free interval				
*De novo* (<3 months)	53	26%	17	34%
3–24 months	32	16%	7	14%
>24 months	118	58%	26	52%
Systemic treatments given for ABC				
Chemotherapy with or without targeted therapy	130	64%	37	74%
Endocrine therapy with or without targeted therapy	124	61%	26	52%
None	21	10%	5	10%
Number of systemic treatment lines given				
Median (range)	2	(0–10)	2	(0–8)
0	21	10%	5	10%
1	54	27%	18	36%
2	31	15%	7	14%
3	23	11%	5	10%
4+	74	36%	15	30%
Final systemic treatment				
Chemotherapy with or without targeted therapy	109	54%	32	64%
Endocrine-targeted therapy	13	6%	12	24%
Endocrine therapy	54	26%	0	0%
Targeted therapy only	6	3%	1	2%
No systemic therapy	21	10%	5	10%
Performance status at start last treatment line				
WHO 0–1	81	40%	18	36%
WHO 2–3	100	49%	28	56%
WHO 4	2	1%	1	2%
Unknown	20	10%	3	6%
Cause of death				
Breast cancer	176	87%	47	94%
Progression of disease	167	82%	39	78%
Complication of treatment for ABC	9	4%	8	16%
Other	13	6%	3	6%
Unknown	14	7%	0	0%
Time between diagnosis ABC and death (years)				
<1	76	37%	21	42%
1–3	97	48%	28	56%
≥4	30	15%	1	2%

ABC, advanced breast cancer; HR, hormone receptor; HER2, Human Epidermal growth factor Receptor 2; WHO, World Health Organization.

**Table 2:** Frequency of health care use in the last six months of life.

Variation	*n* = 203		%	
Systemic treatment near end of life				
New chemotherapy ≤30 days before death	19		9%	
Ongoing chemotherapy ≤14 days before death	42		21%	
Health care use within the last 6 months of life				
ER-visit	144		71%	
Hospital admission (*reason*)	154 *		76%	
Toxicity of systemic therapy		18		12%
Complication of other therapy		6		4%
Tumor related symptoms		92		60%
Other		22		14%
Unknown		35		23%
ICU-admission (reason)	12 **		6%	
Toxicity of systemic therapy		4		33%
Complication of other therapy		1		8%
Tumor related symptoms		6		50%
Other		1		8%
Unknown		3		25%
CPR	3		2%	
Mechanical ventilation	10		5%	
Surgery	9		4%	
Radiotherapy	43		21%	
Place of death				
Hospital (localization)	50		25%	
General ward		40		80%
ICU or Medium care		9		18%
ER		1		2%
Hospice	28		14%	
Rehabilitation	5		3%	
Home	107		53%	
Discharged from hospital, place unknown	13		6%	

* *n* = 19 patients had >1 reason for hospital admission ** *n* = 3 patients had >1 reason for ICU admission.

**Table 3:** Characteristics associated with hospital admission and death in the hospital in the last six months of life, *n* = 200 ^#^.

Characteristic	Category	Hospital Admission ≤6 Months of Life		Death in Hospital	
		Frequency	Multivariable	Frequency	Multivariable
		%	OR (95%CI)*^p^*^-Value^	%	OR (95%CI)*^p^*^-Value^
Incidence year	2007–2009	77	reference	24	reference
	2010–2013	71	0.66 (0.30–1.49)	25	1.49 (0.65–3.40)
	2014–2017	86	1.45 (0.38–5.52)	21	1.14 (0.37–3.84)
Age at diagnosis	<65 years	84	3.12 (1.37–7.07)	27	0.79 (0.34–1.71)
	65+ years	67	reference	21	reference
Subtype	HR+/HER2−	75	reference	25	reference
	HR+/HER2+	62	0.26 (0.09–0.76) **	21	0.59 (0.18–1.90)
	HR−/HER2+	90	1.92 (0.37–9.92)	25	1.15 (0.37–3.95)
	TN	85	1.21 (0.35–4.23)	22	0.51 (0.14–1.81)
Metastatic-free interval	de novo	89	4.41 (1.55–12.5) ***	32	2.03 (0.88–4.72)
	3–24 months	75	0.55 (0.19–1.63)	22	0.92 (0.29–2.91)
	>24 months	70	reference	21	reference
WHO performance status	0–1	73	reference	20	reference
	2–4	78	1.61 (0.76–3.41)	27	1.56 (0.73–3.32)
Survival time	<1 year	81	2.41 (0.97–5.99) *	27	1.01 (0.43–2.37)
	≥1 year	73	reference	23	reference
New chemotherapy ≤30 days before death	No	74	reference	18	reference
	Yes	95	3.70 (0.16–8.33)	79	14.4 (2.85–73.0) ***
Ongoing chemotherapy ≤14 days before death	No	71	reference	17	reference
	Yes	95	12.8 (1.38–120) ***	50	2.05 (0.67–6.28)

^#^ 3 patients with unknown subtype were excluded; * *p* < 0.10; ** *p* < 0.05; *** *p* < 0.01. ABC, advanced breast cancer; CI, confidence interval; HR, hormone receptor; HER2, Human Epidermal growth factor Receptor 2; OR, odds ratio; WHO, World Health Organization.

**Table 4:** Characteristics associated with chemotherapy use near the end of life, *n* = 200 ^#^.

Characteristic	Category	New Chemotherapy ≤30 Days before Death		Ongoing Chemotherapy ≤14 Days before Death	
		Frequency	Multivariable	Frequency	Multivariable
		%	OR (95%CI)*^p^*^-Value^	%	OR (95%CI)*^p^*^-Value^
Incidence year	2007–2009	14	reference	22	reference
	2010–2013	8	0.39 (0.13–1.19)	20	0.81 (0.36–1.83)
	2014–2017	3	0.13 (0.01–1.21)	24	1.10 (0.35–3.43)
Age at diagnosis	<65 years	16	11.8 (2.46–56.9) ***	32	4.76 (2.04–11.1) ***
	65+ years	2	reference	9	reference
Subtype	HR+/HER2−	8	reference	16	reference
	HR+/HER2+	10	0.64 (0.14–2.87)	24	1.17 (0.41–3.36)
	HR−/HER2+	5	0.45 (0.05–4.08)	15	0.71 (0.18–2.81)
	TN	19	1.58 (0.40–6.18)	44	3.65 (1.36–9.80) **
Metastatic-free interval	de novo	9	1.24 (0.35–4.39)	21	1.11 (0.46–2.69)
	3–24 months	13	0.68 (0.16–2.91)	22	0.66 (0.22–1.95)
	>24 months	9	reference	21	reference
WHO performance status	0–1	9	reference	18	reference
	2–4	10	1.68 (0.56–4.99)	24	1.50 (0.70–3.22)
Survival time	<1 year	15	4.50 (1.40–14.5) ***	24	1.49 (0.66–3.40)
	≥1 year	6	reference	19	reference

^#^ 3 patients with unknown subtype were excluded; ** *p* < 0.05; *** *p* < 0.01. ABC, advanced breast cancer; CI, confidence interval; HR, hormone receptor; HER2, Human Epidermal growth factor Receptor 2; OR, odds ratio; WHO, World Health Organization.

## Data Availability

Data may be available on request, from the corresponding author.

## Supplementary Materials

The following are available online at https://www.mdpi.com/article/10.3390/cancers13215271/s1, Table S1: Final systemic treatment choices before death (*n* = 203).
