# Supplementary material for: Healthcare Use during the Last Six Months of Life in Patients with Advanced Breast Cancer
Source: Cancers (Basel). 2021 Oct 20;13(21):5271. doi: 10.3390/cancers13215271 (PMC8582356; doi:10.3390/cancers13215271)
Supplement: Supplementary file 1 [file cancers-13-05271-s001.zip › cancers-1413417-supplementary.pdf]

Supplementary Table S1. Final systemic treatment choices before death (n=203).

**Final Systemic Treatment**		
**Chemotherapy with or without targeted therapy**	**109**	**54%**
Capecitabine	27	
Taxane	19	
Chemotherapy plus HER2-targeted therapy	17	
Paclitaxel plus bevacizumab	12	
CMF	12	
Eribulin	6	
AC-containing regimens	8	
Vinorelbine	4	
Gemcitabine	1	
Carboplatin	1	
Other chemotherapy combinations	2	
**Endocrine-targeted therapy**	**13**	**6%**
Everolimus plus exemestane	9	
Endocrine therapy plus trastuzumab	2	
Endocrine therapy plus bevacizumab	1	
Endocrine therapy plus study medication	1	
**Endocrine therapy**	**54**	**26%**
Aromatase inhibitors	25	
Fulvestrant	15	
Tamoxifen	9	
Megestrol	5	
**Targeted therapy only**	**6**	**3%**
Trastuzumab	2	
Palbociclib	1	
Brivanib	3	
**No systemic therapy**	**21**	**10%**
